# Evolution of Telepathology: A Comprehensive Analysis of Global Telepathology Literature Between 1986 and 2017

**DOI:** 10.5146/tjpath.2019.01484

**Published:** 2020-09-15

**Authors:** Engin Şenel, Yılmaz Baş

**Affiliations:** Department of Dermatology, Hitit University Faculty of Medicine, Çorum, Turkey; Department of Pathology, Hitit University Faculty of Medicine, Çorum, Turkey

**Keywords:** Telepathology, Telemedicine, Bibliometrics, Scientometrics, Publication trend

## Abstract

***Objective:*** Telepathology is an application of telemedicine providing remote evaluation and consultation of digital pathology images and can be used for educational or experimental purposes. Bibliometrics is a statistical discipline investigating publication patterns and trends in a certain academic field. Although bibliometric and scientometric studies are becoming increasingly popular, the relevant literature contains only one limited article related to telepathology. The aim of our study was to perform a holistic bibliometric analysis of the telepathology literature.

***Material and Method:*** Since the first article on telepathology was published in 1986, we included all indexed articles retrieved from Web of Science databases between 1986 and 2017.

***Results:*** We found that the USA covering 43.01% of all literature was the leading country in the telepathology field and was followed by Germany, Italy and the UK (n=120, 90 and 83, respectively). The countries with the most contributions were located in the continents of Europe and North America. The most productive source titles were Human Pathology, Journal of Telemedicine and Telecare, and Modern Pathology. Harvard University ranked first with 59 articles. The most commonly used keywords of the telepathology literature were “telepathology”, “telemedicine”, “digital pathology”, “virtual microscopy” and “telecytology”. We noted that all of the ten countries with the most contributions were in the developed category of UN classification and all twenty of the most productive institutions were from developed countries.

***Conclusion:*** We suggest that researchers from developing and least developed countries should be encouraged to carry out novel studies since telemedicine is a required and promising technology for rural developing or least developed areas in which access to health care is difficult.

## INTRODUCTION

Telemedicine is the process of transmitting medical information and the application of medical services using communication technologies (1). The World Health Organization (WHO) defines telemedicine as *“..the delivery of healthcare services, where distance is a critical factor, by all healthcare professionals using information and communication technologies for the exchange of valid information for diagnosis, treatment and prevention of disease and injuries, research and evaluation, and for the continuing education of healthcare providers, all in the interests of advancing the health of individuals and their communities*…” (2). Telepathology is a relatively novel practice of telemedicine providing remote evaluation of digital pathology images. Telepathology is defined by the American Telemedicine Association as “*the electronic multimedia communication across a network of pathology-related information, between two or more locations for use between pathologists and/or qualified laboratory personnel, and may include involvement by clinicians and/or patients*” (3). Telepathology can be used for educational or experimental purposes, primary diagnosis and secondary consultation in all divisions of pathology including histo-cytopathology, autopsy, and surgical and anatomical pathology (4).

Bibliometrics provides holistic data on publication trends and patterns and is described as “science of science” (5). Although scientometric and bibliometric studies have been popular recently, only one study relevant to telepathology has been reported so far to the best of our knowledge. To fully utilize the advantages of telepathology, individual feasibility studies are necessary, as data analysis, organizations, societies and infrastructures (6). Our study aims to present a comprehensive bibliometric analysis of the literature of telepathology.

## MATERIAL and METHOD

All data of our study was retrieved from four databases of Web of Science (WOS, Thomson Reuters, New York, NY, USA) titled Web of Science Core Collection, Korean Journal Database, SciELO Citation Index and Russian Science Citation Index. We used the keywords of “telepathology” and “telecytology” to search the WoS database. All documents produced between 1986 and 2017 were included. Since the first article of telepathology literature was published in 1986, we chose 1986 as the starting year. We used GunnMap to generate a global productivity map (7). VOSviewer was the freeware tool used for creating scientometric networks in our study (8). We used the United Nations (UN) system for country classification (9).

## RESULTS

### General Feature of the Literature

**Figure 1:**
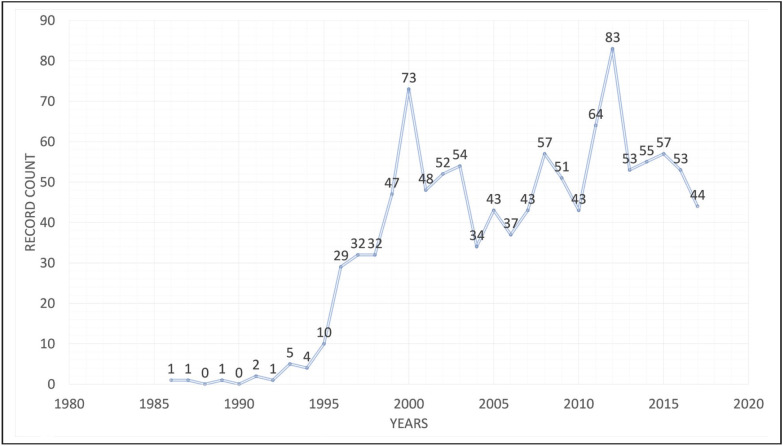
Number of telepathology publications by year.

Our search yielded 1109 articles in the telepathology literature between 1986 and 2017. Only 237 documents (21.37%) were open access. The peak year for number of publications was 2012 with 83 articles. No pattern was detected in the progress of the total number of publications by year. No items were produced in the years of 1988, 1990 and 1992 (Figure 1). English is the main language of the literature (94.91%) followed by German and French (2.43 and 2.08%, respectively). The most studied research areas were found to be health care sciences, telecommunications, pathology, computer science and oncology (68.89, 63.12, 55.45, 42.38 and 39.58%, respectively; Table 1). The most common document types in the literature were original article, meeting abstract and review (72.77, 28.40 and 18.12%, respectively; Table 2).

**Table 1:** The most studied research areas of the telepathology literature between 1986 and 2017.

**Research Area**	**Record Count**	**% of 1109 articles**
Health Care Sciences	764	68.89
Telecommunications	700	63.12
Pathology	615	55.45
Computer Science	470	42.38
Oncology	439	39.58
Mathematical Computational Biology	370	33.36
Microscopy	326	29.39
Medical Laboratory Technology	300	27.05
Science Technology	297	26.78
General Internal Medicine	279	25.15
Radiology	278	25.07
Surgery	247	22.27
Imaging Science	227	20.47
Engineering	205	18.48
Medical Informatics	197	17.76
Cell Biology	188	16.95
Information Science	143	12.89
Dermatology	126	11.36
Educational Research	126	11.36
Mathematics	122	11.01
**Total**	**1109**	**100**

**Table 2:** Document types published in the telepathology literature.

**Document Types**	**Record Count**	**% of 1109 articles**
Original Article	807	72.77
Meeting Abstract	315	28.40
Abstract	201	18.12
Review	147	13.25
Letter	52	4.69
Editorial	38	3.43
Clinical Trial	23	2.07
News	8	0.72
Case Report	7	0.63
Correction	3	0.27
Book	2	0.18
Reference Material	2	0.18
Biography	1	0.09
Unspecified / Other	358	32.28
**Total**	**1109**	**100**

### Productivity of Countries, Authors, Journals and Institutions

**Figure 2:**
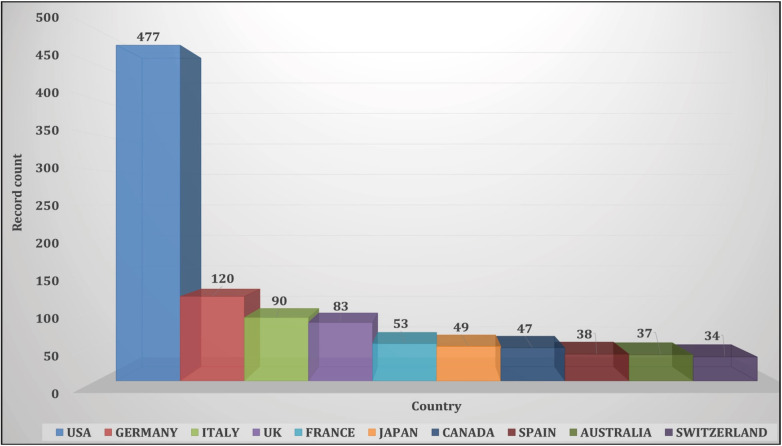
Top ten countries publishing telepathology articles between 1986 and 2017.

The USA, covering 43.01% of the literature, was detected to rank first among countries with 477 articles, followed by Germany, Italy and the UK (n=120, 90 and 83, respectively; Figure 2). China stood out among developing countries according to the UN classification with 18 articles (3.07%) followed by Brazil, India and Colombia (1.44, 1.26 and 0.81%, respectively). From the underdeveloped (least developed) countries, Benin, Burundi, Congo, Rwanda, Senegal, Sudan and Zambia produced one article each.

**Figure 3:**
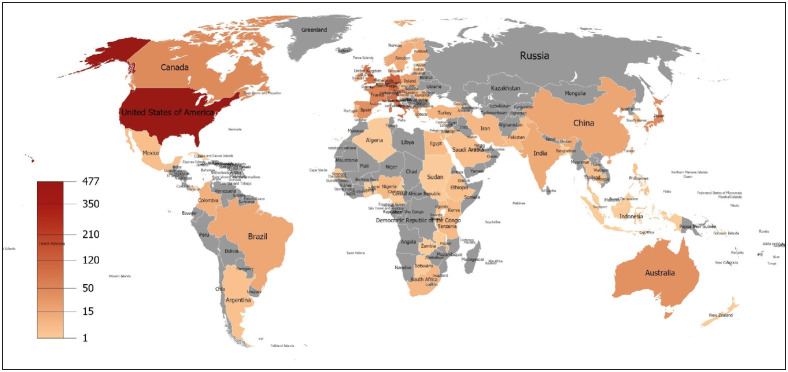
Publication density of world countries in the telepathology field.

The countries contributing the most countries were located in the continents of Europe and North America (Figure 3). Weinstein RS was the most prolific author with 44 papers (3.97%, Table 3). The source titles with the most contributions were *Human Pathology*, *Journal of Telemedicine and Telecare *and *Modern Pathology* (n= 80, 69 and 56 articles, respectively; Table 4). The most productive meeting in this field was the *10th European Congress on Telepathology *and the *4th International Congress on Virtual Microscopy* with 20 proceedings (Table 5). Harvard University was the leading institution with 59 documents followed by the University of Arizona, Pennsylvania Commonwealth System of Higher Education, and the University of Pittsburgh (n= 52, 50 and 48 items, respectively; Table 6).

**Table 3:** The 20 most prolific authors of the telepathology literature between 1986 and 2017.

**Author**	**Record Count**	**%**
Weinstein RS	44	3.97
Della Mea V	29	2.61
Krupinski EA	28	2.52
Kayser K	27	2.43
Pantanowitz L	23	2.07
Beltrami CA	19	1.71
Kayser G	18	1.62
Evans AJ	16	1.44
Giansanti D	16	1.44
Graham AR	16	1.44
Parwani AV	16	1.44
Yagi Y	16	1.44
Oberholzer M	15	1.35
Giovagnoli MR	14	1.26
Bhattacharyya AK	13	1.17
Hufnagl P	13	1.17
Schrader T	13	1.17
Tetu B	13	1.17
Brauchli K	12	1.08
Soyer HP	12	1.08

**Table 4:** The journals with the most contributions to the telepathology literature.

**Journals**	**Record Count**	**% of 1109 items**
Human Pathology	80	7.21
Journal of Telemedicine and Telecare	69	6.22
Modern Pathology	56	5.05
Laboratory Investigation	53	4.78
Diagnostic Pathology	49	4.42
Archives of Pathology & Laboratory Medicine	42	3.79
Analytical Cellular Pathology	37	3.34
Telemedicine Journal and e-Health	35	3.16
Studies in Health Technology and Informatics	28	2.52
American Journal of Clinical Pathology	27	2.43
Histopathology	25	2.25
Virchows Archiv	21	1.89
Journal of Clinical Pathology	19	1.71
Journal of Pathology	19	1.71
Acta Cytologica	12	1.08
Journal of the American Academy of Dermatology	12	1.08
Pathology - Research and Practice	12	1.08
Diagnostic Cytopathology	11	0.99
Der Pathologe	10	0.90
Annales de Pathologie	9	0.81

**Table 5:** The meetings or conferences in which the most documents on telepathology were presented.

**Meeting Titles**	**Record Count**	**% of 37283**
10th European Congress on Telepathology and 4th International Congress on Virtual Microscopy	20	1.80
102nd Annual Meeting of The United States and Canadian Academy of Pathology	14	1.26
101st Annual Meeting of The United States and Canadian Academy of Pathology	10	0.90
106th Annual Meeting of The United States and Canadian Academy of Pathology	9	0.81
92nd Annual Meeting of The United States and Canadian Academy of Pathology	8	0.72
97th Annual Meeting of The United States and Canadian Academy of Pathology	8	0.72
9th European Congress on Telepathology and 3RD International Congress on Virtual Microscopy	8	0.72
103rd Annual Meeting of The United States and Canadian Academy of Pathology	6	0.54
104th Annual Meeting of The United States and Canadian Academy of Pathology	6	0.54
11th International Conference on Informatics Management and Technology in Healthcare	6	0.54

**Table 6:** The 20 most productive institutions in the telepathology literature between 1986 and 2017.

**Institutions**	**Country**	**Record Count**	**%**
Harvard University	USA	59	5.32
University of Arizona	USA	52	4.69
Pennsylvania Commonwealth System of Higher Education	USA	50	4.51
University of Pittsburgh	USA	48	4.33
VA Boston Healthcare System	USA	42	3.79
Free University of Berlin	Germany	34	3.07
Charité Medical University of Berlin	Germany	32	2.89
Humboldt University of Berlin	Germany	32	2.89
Massachusetts General Hospital	USA	29	2.61
University of California System	USA	29	2.61
University of Udine	Italy	29	2.61
United States Department of Defense	USA	25	2.25
Assistance Publique - Hôpitaux de Paris	France	21	1.89
University of Toronto	Canada	21	1.89
University of Basel	Switzerland	20	1.80
University of London	UK	18	1.62
Johns Hopkins University	USA	17	1.53
University Health Network	Canada	17	1.53
Cedars-Sinai Medical Center	USA	16	1.44
Istituto Superiore di Sanita	Italy	16	1.44

### Citations, Keywords and Bibliometric Network Analyses

The h-index is a calculated metric value to measure productivity and citation impact in a certain area. The h-index of telepathology literature was 52. The total number of citations was 14,911 (8,997 without self-citations) and the average number of citations per item were 13.45.

The most cited article was an original article titled “*Telemedicine Technology and Clinical Applications*” published in 1995 by Perednia and Allen (Table 7). This article did not only focus on telepathology and mentioned telepathology as an application of store-and-forward telemedicine (10). The most cited document focusing on telepathology was an original article titled “*Overview of telepathology, virtual microscopy, and whole slide imaging: prospects for the future*” published in 2009 by Weinstein RS who is the most prolific author of telepathology literature, and was cited 146 times. The article presented information relevant to fourth-generation telepathology systems, so-called virtual slide telepathology systems used for educational purposes (11).

**Table 7:** The most cited articles in telepathology literature by decades.

**Article**	**Author(s)**	**Year**	**Total Citation**	**Average Citations per Year**
Telemedicine Technology and Clinical-Applications	Perednia, DA; Allen, A	1995	387	15.48
Overview of Telepathology, Virtual Microscopy, and whole Slide Imaging: Prospects for the Future	Weinstein, Ronald S.; Graham, Anna R.; Richter, Lynne C.; *et al.*	2009	149	13.55
Validating whole Slide Imaging for Diagnostic Purposes in Pathology Guideline from The College of American Pathologists Pathology and Laboratory Quality Center	Pantanowitz, Liron; Sinard, John H.; Henricks, Walter H.; *et al.*	2013	131	18.71
Eye-Movement Study and Human Performance Using Telepathology Virtual Slides. Implications for Medical Education and Differences with Experience	Krupinski, Elizabeth A.; Tillack, Allison A.; Richter, Lynne; *et al.*	2006	125	8.93
Critical Comparison of 31 Commercially Available Digital Slide Systems in Pathology	Garcia Rojo, Marcial; Bueno Garcia, Gloria; Peces Mateos, Carlos; *et al.*	2006	122	8.71
Robust Segmentation of Overlapping Cells in Histopathology Specimens Using Parallel Seed Detection and Repulsive Level Set	Qi, Xin; Xing, Fuyong; Foran, David J.; *et al.*	2012	121	15.13
Telepathology Overview: From Concept to Implementation	Weinstein, RS; Descour, MR	2012	119	6.26
Telepathology and the Networking of Pathology Diagnostic Services	Weinstein, RS; Bloom, KJ; Rozek, LS	1987	111	3.36
Digital pathology: current status and future perspectives	Al-Janabi, Shaimaa; Huisman, Andre; Van Diest, Paul J.	2012	110	13.75
Telepathology: A ten-year progress report	Weinstein, RS; Bhattacharyya, AK; Graham, AR; *et al.*	1997	109	4.74

**Figure 4:**
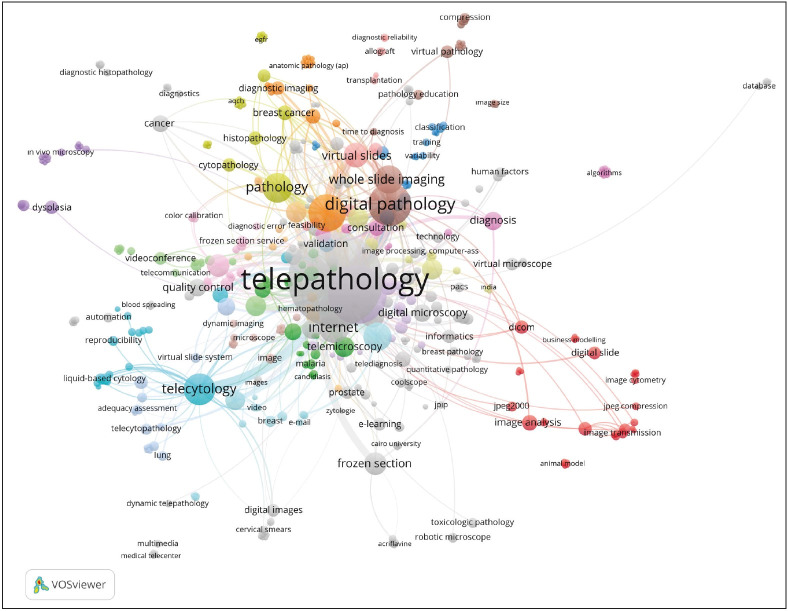
Bibliometric network of the most used keywords in the telepathology literature.

**Figure 5:**
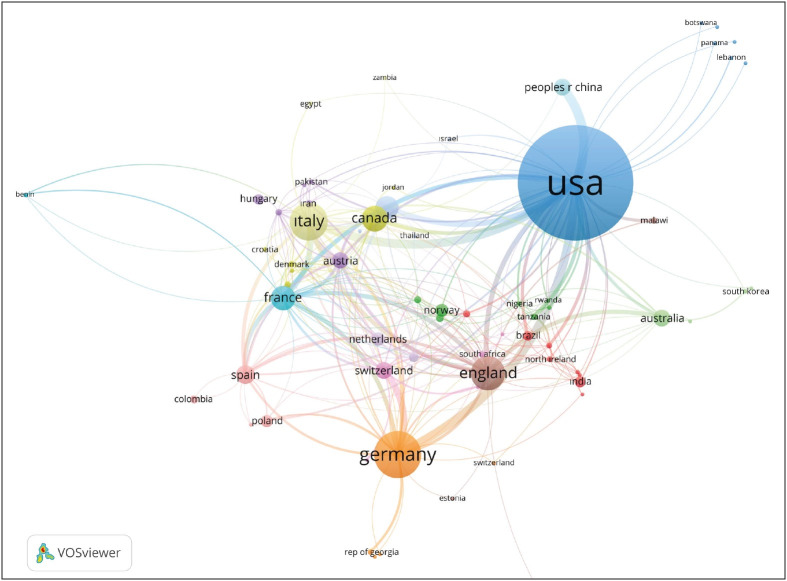
The most collaborative countries in the telepathology literature.

The most common keywords of the telepathology literature were “telepathology”, “telemedicine”, “digital pathology”, “virtual microscopy” and “telecytology” (Table 8). Bibliometric network analysis of the keywords revealed a “starburst pattern” in which keywords of “telepathology”, “digital pathology” and “internet” were centered (Figure 4). As we analyzed the co-authorship among countries, it was found that the most collaborative countries were the USA with 377 documents and 6286 citations (total link strength, TLS=127) followed by Italy, the UK and Germany (TLS=79, 73 and 71, respectively; Figure 5).

**Table 8:** The 20 most used keywords in the telepathology literature.

**Keyword (total link strength)**	
1 Telepathology (842)	11 Frozen section (38)
2 Telemedicine (333)	12 Telehealth (64)
3 Digital pathology (181)	13 Telemicroscopy (49)
4 Virtual microscopy (181)	14 Surgical pathology (46)
5 Telecytology (132)	15 Teleradiology (39)
6 Internet (90)	16 Cytology (63)
7 Pathology (119)	17 Digital imaging (44)
8 Whole-slide imaging (96)	18 Diagnosis (46)
9 Virtual slide(s) (89)	19 Quality assurance (39)
10 Diagnostic accuracy (53)	20 Digital microscopy (35)

## DISCUSSION

The earliest telemedicine applications date back to the eighteenth and nineteenth centuries. In these centuries, patients wrote detailed letters containing medical histories and symptoms and sent them to specialist doctors by couriers, and doctors responded with the diagnosis, treatment plan and prescription (12). Willem Einthoven (1860-1927), a Dutch doctor and physiologist who invented the first electrocardiogram (ECG) transmitted ECGs of the patients from the hospital to an off-site laboratory by means of telegraph (13). The first remote assessment of medical images, reported in 1950, was performed between the Chester County Hospital and Philadelphia over a distance of twenty-eight miles via commercial telephone wires or radio and described as “telognosis” (14). In the late 1950s, National Aeronautics and Space Administration (NASA) initiated the Space Technology Applied to Rural Papago Advanced Health Care (STARPAHC) Project for telemedical consultation of people living in remote locations of Arizona’s Papago Indian Reservation with little or no medical services. In 1964, a closed circuit television system was introduced between the Nebraska Psychiatric Institute and the Norfolk State Hospital 112 miles away to use telemedicine for neurologic and psychiatric evaluation of the patients (15).

The first recorded telepathology procedure was performed in the late 1960s between Massachusetts General Hospital and Logan Airport Medical Station in Boston via a real-time “television microscopy” service (16). Since this onset, the research area of telepathology has been growing into subfields. The first paper in the telepathology field was an editorial published in 1986 and titled “Prospects for Telepathology” by Ronald Weinstein. The author defined telepathology as “the practice of pathology by visualizing an indirect image on a television screen rather than viewing a specimen directly through a microscope…”. Interestingly, there was no study on telepathology in the medical literature prior to this definition. Weinstein predicted the future of computer technology perfectly and defined the level of development of telepathology at that time as “an embryonic stage of development” (16). Over the last 30 years, the generation of telepathology systems has improved rapidly with the evolution of digital microscopy: video-microscopy, robotic microscopy and the upgrade to virtual slide processing systems. This change is a revolution in the traditional pathology practice in today’s computer age (17). Increased workload, case complexity, financial constraints, and staffing shortages justify wider implementations of digital pathology (6). The possibility to consult expert colleagues using telepathology, particularly for cases not requiring molecular investigations in external laboratories, may also permit significant financial savings, reduce the turnaround time, facilitate the international change of information, and support the sharing of even more cases (18).

Bibliometrics is a popular statistical application providing quantitative and qualitative analysis of a certain academic field (19). The term was coined by Alan Pritchard in the late 1960s as ‘‘an application of mathematics and statistical methods to books and other media of communication” although Siyali Ramamrita Ranganathana, who was a librarian and mathematician from India, created the principal ideas behind bibliometrics and scientometrics in the 1940s (19,20). Bibliometric studies have become increasingly popular in the academic literature in the last decades, and a total of 8806 reports have been published in the time period of our study according to the WoS databases. Although the term of telepathology has been included in MeSH terminology since 1996, the scientific literature contained only one bibliometric study of telepathology. Della Mea reported the first and only bibliometric study in the telepathology literature in 2011 (21). The data of this study was extracted from PubMed and only 967 papers published in 344 different journals from 34 countries were detected between 1986 and 2010. The USA was found to be the leading country as noted in our study with 310 articles (32.06%) followed by Germany, Italy, UK and Japan (n= 81, 46, 40 and 27 documents, respectively). The major journals in the telepathology field were *Journal of Telemedicine and Telecare*, *Human Pathology* and the *Telemedicine Journal* (n= 71, 65 and 27 items, respectively) (21).

In conclusion, we found that all ten of the most contributing countries were in developed category of UN classification although telemedicine is a required and promising technology for rural developing or least developed areas in which access to health care is difficult. Also, all twenty most productive institutions were from developed countries. We suggest that researchers from developing and least developed countries should be encouraged to carry out novel studies.

## Conflict of Interest

The authors declare no conflict of interest.
